# Anterior deflected urinary stream in female children: description of a unique clinical entity and surgical management

**DOI:** 10.3389/fped.2024.1434021

**Published:** 2024-09-25

**Authors:** Matthew S. Swallow, Cynthia A. Sharadin, Anthony J. Schaeffer, Deborah L. Jacobson, Glen A. Lau

**Affiliations:** Division of Urology, Department of Surgery, University of Utah/Primary Children’s Hospital, Salt Lake City, UT, United States

**Keywords:** pediatric urology, urethra, lower urinary tract symptoms, surgery, bladder

## Abstract

**Objectives:**

To investigate the clinical presentation and outcomes for a series of female pediatric patients with severe anterior deflection of the urinary stream (ADUS) who were managed via urethromeatoplasty.

**Methods:**

This single institution retrospective cohort study used the institutional billing database to identify female patients ≤18 years who received a urethromeatoplasty (CPT 53450) from 2007 to 2022. Patients were included if a substantial anterior deflection of their urinary stream was the primary indication for surgery. Patients were excluded if they were >18 years of age, had a history of prior genital trauma, or underwent surgery for an indication other than a deflected urinary stream.

**Results:**

Twenty female patients underwent urethromeatoplasty between 2007 and 2022, with a median age of 3 years old. All patients presented with difficulty aiming the urinary stream during toilet training and demonstrated a web of tissue along the posterior aspect of the urethral orifice. 19/20 patients noted immediate response (i.e., normal, non-deflected urinary stream) after the urethromeatoplasty with no further urinary complaints. There were no post-operative complications within a 90-day period.

**Conclusions:**

ADUS is a clinical entity characterized by a web of deflecting tissue at the female posterior urethral meatus that causes severe urinary deflection without other urologic symptoms. This is not well-described in the literature. Surgical correction via urethromeatoplasty is safe and effective.

## Introduction

Deflected urinary stream in boys is a relatively common occurrence, typically due to meatal stenosis after circumcision. Boys with meatal stenosis are often noted to have anterior deflection of the urinary stream, most frequently with few lower urinary tract symptoms (1). The preferred treatment is surgical meatotomy, which has a high success rate (2).

Abnormalities of the urethral orifice in female children are significantly less common. These abnormalities are often identified after infancy in female children are typically accompanied with symptoms of voiding dysfunction and sequelae such as vesicoureteral reflux or intermittent urinary tract infections (3–5). Labial adhesions are a frequent cause of recurrent urinary tract infections (UTIs) and voiding dysfunction (6), but urethral abnormalities are less common. Anterior deflected urinary stream (ADUS) associated with dysfunctional voiding has previously been described in female children, and surgical correction via meatoplasty in patients with symptoms of lower urinary tract dysfunction and concomitant ADUS has been shown to be more effective than conservative management with behavioral therapy (7, 8). However, there is limited data available regarding the treatment of patients with ADUS without symptoms of dysfunctional voiding.

Our study aims to describe the presentation, anatomic abnormality, and surgical treatment of female patients with monosymptomatic ADUS through an analysis of our institution's surgical records. We hypothesized that patients who underwent urethromeatoplasty for monosymptomatic ADUS would have low rates of surgical complications and high rates of treatment success.

## Methods

We conducted a retrospective analysis within our institution's electronic medical records (EMR) from 2007 to 2022 for female pediatric patients who underwent a urethromeatoplasty (CPT 53450). All patients who underwent urethromeatoplasty were due to a chief complaint of a severely anteriorly deflected urinary stream discovered during potty training (urinary stream exiting the toilet) that were not resolved with conservative treatment via positional adjustments while voiding on the toilet. Patients were excluded if they were >18 years of age, had a history of prior genital trauma, or underwent surgery for an indication other than a deflected urinary stream.

We collected descriptive data from the EMR, including patient demographics, presenting symptoms, intraoperative exam findings, procedure performed, 90-day postoperative complications, and reported results in follow-up. The EMR was also searched for potential etiologies of anterior deflected stream such as a history of vulvovaginitis, labial adhesions, genital Stevens-Johnson syndrome, sexual abuse, prior trauma, or recurrent urinary tract infections.

**Figure 1:**
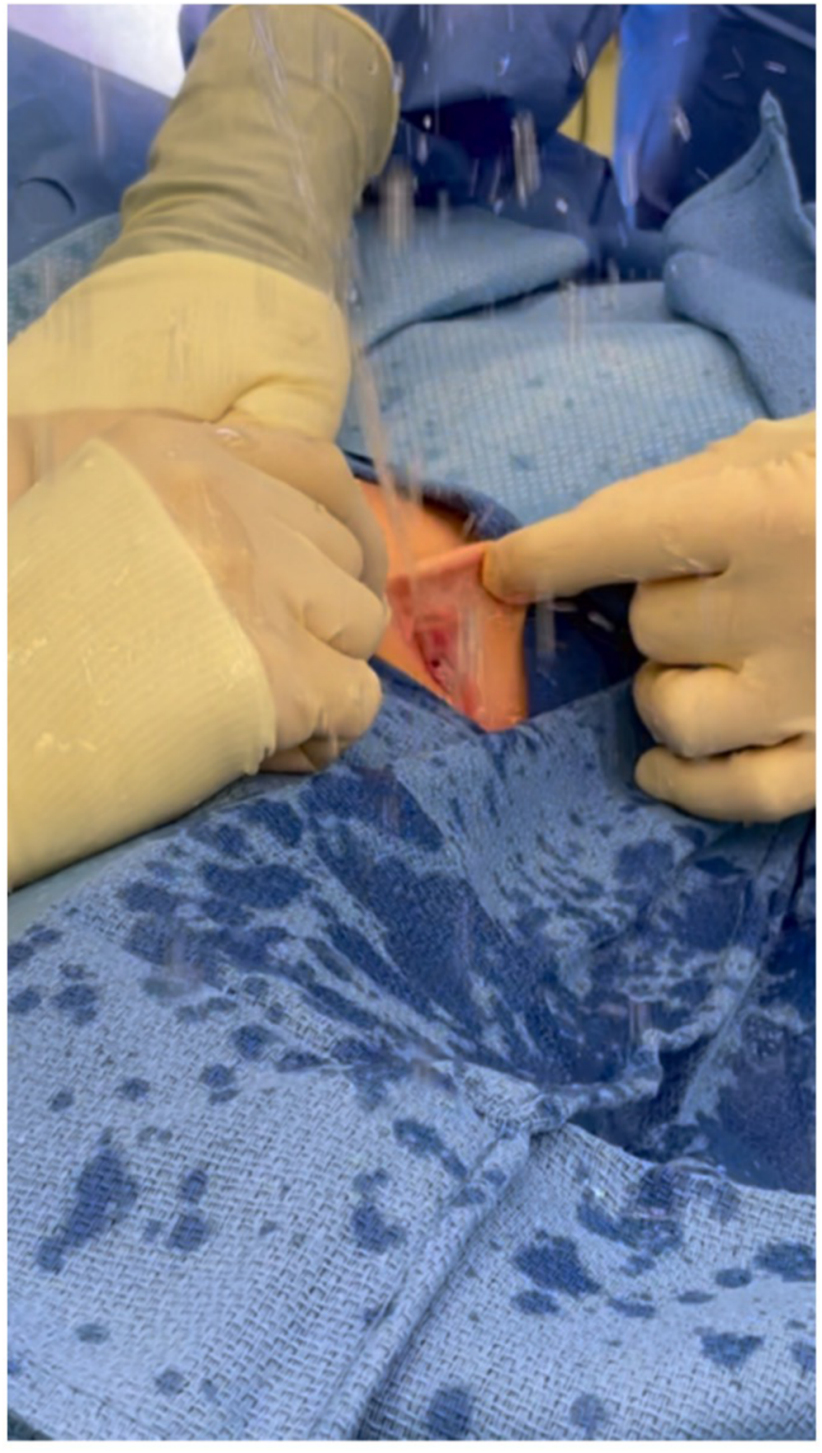
Intraoperative Crede maneuver to confirm ADUS.

**Figure 2:**
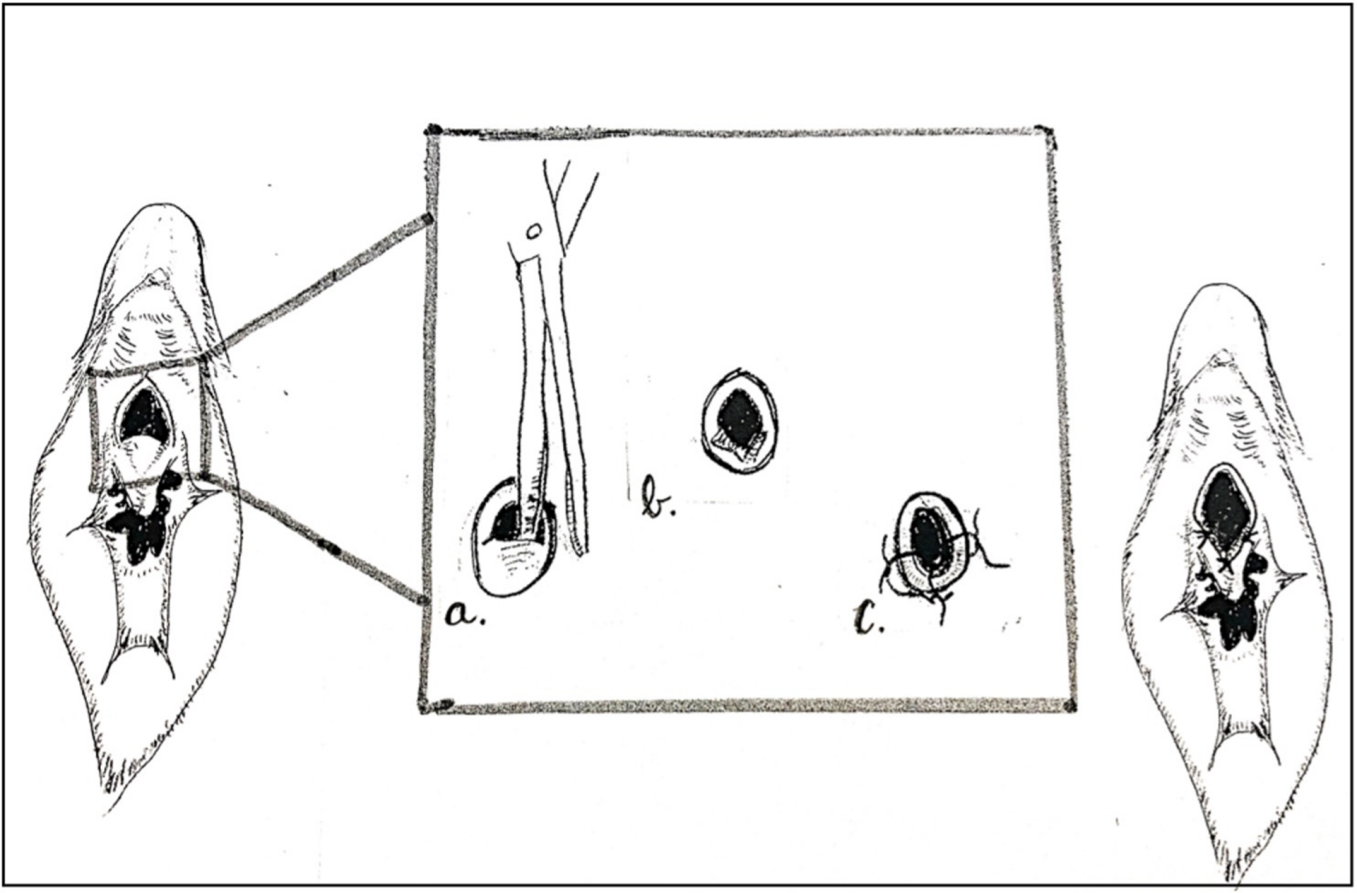
Surgical technique for female urethromeatoplasty. **(a)** Clamp then incise along the midline of the posterior meatal flap. **(b)** Incised flap, which can be excised if significant redundancy. **(c)** Mucosal advancement interrupted sutures placed at lateral and posterior borders.

Urethromeatoplasty was performed in the operating room under general anesthesia. The introitus and urethra were inspected with optional cystourethroscopy (not routinely performed as some surgeons felt this was unnecessarily invasive). A Crede maneuver to confirm ADUS was typically performed (Figure 1). ADUS caused by a posterior web of tissue was then corrected by crushing the flap of posterior tissue with a straight clamp. The crushed tissue was then incised using scissors. 5-0 absorbable interrupted sutures (Monocryl or Chromic Catgut depending on surgeon preference) were then placed to reapproximate the divided tissue posteriorly and laterally (Figure 2). A foley catheter was not placed post-operatively.

## Results

Twenty-two female patients who underwent urethromeatoplasty between 2007 and 2022 were identified within the Primary Children's Hospital EMR. Two patients were excluded from the study (one for a history of prior genital trauma, one for age >18 years).

The median age for the population was 3 years old [IQR 2.75, 3.25 years; min 2, max 5 years]. The only presenting symptoms for these patients was substantial difficulty aiming the urinary stream into the toilet bowel during toilet training. No patient had a history of another genital abnormality (such as labial adhesions, cystic vulvovaginal masses, or other introital abnormalities), bowel and bladder dysfunction (lower urinary tract and bowel dysfunction without an identifiable or recognizable neurologic abnormality) (9), or a history of urinary tract infections. Uroflow studies were not possible because the females were unable to aim their urine stream into the device. Preoperative urinalysis, urine culture and renal/bladder imaging was not obtained given the only preoperative complaint was a deflected stream. All 20 of the patients who underwent urethromeatoplasty were found to have a mucosal web of tissue along the posterior edge of the meatus. Patient demographics are summarized in Table 1.

**Table 1:** Patient demographics.

Age, median years (IQR)	3 (2.75–3.25)
Chief complaint, ADUS, *N* (%)	20 (100)
Other genital abnormality, *N* (%)	0 (0)
Other congenital abnormality, *N* (%)	0 (0)
History of bladder or bowel dysfunction, *N* (%)	0 (0)
History of recurrent urinary tract infections, *N* (%)	0 (0)
Web of non-obstructing tissue along the posterior aspect of the urethral orifice	20 (100)

There was no strict follow up protocol after the surgery, and thus the majority of patients were instructed to follow up only if there was noted persistence of a deflected urinary stream or if they were having any post-operative concerns. Only one of the twenty patients reported continued deflected urinary stream for a success rate of 95% (19/20). There were no reported complications within a 90-day period (urinary tract infection, bleeding, pain, or presentation to the ER) from any of the study cohort.

## Discussion

Female urethral orifice abnormalities are rare especially among children. Here, we describe severe anterior deflection of the urinary stream in young girls that either prevents or makes potty training especially challenging. There is limited description in the literature regarding the cause and management of ADUS. Our findings suggest that ADUS is caused by a thin web of mucosal tissue along the posterior aspect of the female urethral orifice. This does not necessarily lead to urinary obstruction, but rather acts to deflect the urinary stream anteriorly. Our study population all presented for consultation after this urinary deflection resulted in difficulty with toilet training (i.e., inability to direct the urinary stream into the toilet bowl). This presentation should be distinguished from the patient with anatomic urinary obstruction, who may present lower urinary tract symptoms, recurrent urinary tract infections, or worsening renal function.

There are a few published studies that evaluate management of female children with ADUS who present with other lower urinary tract symptoms. Klijn et al. suggests that many young girls with dysfunctional voiding have underlying ADUS (7). Their study also suggests surgical meatotomy carries a 50% success rate (relief of all urinary symptoms) for children with dysfunction voiding and ADUS (7). Apoznanski et al. studied a group of young girls with detrusor overactivity and found that those who underwent surgical meatotomy for underlying ADUS in addition to oxybutynin were more likely to find relief from urinary tract infections, daytime urinary incontinence, and nocturnal bedwetting compared to patients with a normal urinary stream who were treated with only oxybutynin (8). Our patient population, however, differs in that the children within our study presented with symptoms of ADUS without other urinary complaints. This clinical entity is less studied, with only a single case report by Abbas et al. in which a 3-year-old girl underwent division of a meatal shelf causing monosymptomatic ADUS with good post-operative results (10).

Our success rate after undergoing urethromeatoplasty was 95%, suggesting urethromeatoplasty is highly effective at correcting monosymptomatic ADUS. The one patient with persistent urinary stream issues returned nineteen months post-operatively with complaints of continued anteriorly deflected urinary stream. Although this was somewhat improved, the patient still was having difficulty aiming the urinary stream into the toilet. Unfortunately, the patient was never observed urinating and no video of her urinary stream was obtained. It was hypothesized that the patient would benefit from further division of meatal tissue. The patient was offered a repeat exam under anesthesia with potential repeat meatoplasty, but the patient's family declined repeat surgery and has had no further follow up with our institution.

Urethromeatoplasty to correct ADUS also appears to be both safe and practical. No patients in our study population who underwent surgical correction of ADUS had a known complication. All of the surgeries were done as an outpatient with approximately 20 min of operative time. We recommend the procedure be completed in the operating room under general anesthesia in order to achieve adequate urethral explore. Based on our cohort, however, cystourethroscopy did not identify any abnormalities, and we do not think it is necessary in this patient population.

This study should be viewed in light of its limitations. The low case numbers prevent a more in-depth study of the condition and could lead to missing potential etiologic factors and mask potential postoperative issues. A larger and multi-institutional study would potentially provide more generalizable results. Another limitation would be the retrospective nature of the study and that the study population was generated by analyzing operating room records. Since our entire study cohort underwent urethromeatoplasty, we are unable to compare those with ADUS who are managed with surgical vs. conservative management. A prospective study comparing the results of operative and conservative management for those with ADUS would be valuable. However, given the high success rate, low complication rate, and overall low morbidity of the procedure, we demonstrate that operative management of severe ADUS provides a safe, effective, and rapid cure of the condition.

## Data Availability

The raw data supporting the conclusions of this article will be made available by the authors, without undue reservation.
